# Some Practical Aspects of Modern Medical Science1Read at the annual meeting of the Medical Association of Central New York, at Rochester, October 20, 1896.

**Published:** 1897-04

**Authors:** John O. Palmer

**Affiliations:** Auburn, N. Y. 18 William Street


### SOME PRACTICAL ASPECTS OF MODERN MEDICAL SCIENCE.[1]

By JOHN O. PALMER, M. D., Auburn, N. Y.

I HOPE you will not expect from my title a discussion of the deep scientific questions of the day. My aim is only to rest your minds for a few moments, from the scholarly efforts preceding me, by a consideration of some of the more homely aspects of our work. I shall take the liberty also of rambling to some extent over the fields adjacent to strictly professional limits, and possibly touch upon topics which some may consider non-medical, but which it appears to the writer fall within the province of our profession in its broad comprehensiveness.

In making a doctor, the first step is to make a man, for without the material found only in the gentleman or gentlewoman, the manufacture of a true physician is an impossibility, however well equipped the factory may be through which he or she is advanced. It is morally certain that the fundamental principles of true character will come to the surface and control the conduct in the social economy, as it is physically certain that our material frames will be resolved into their component parts, so fulfilling the inflexible laws of nature in both instances.

The dominion given to the final evolution of creative power, when at the close of the Creator's fiat week he said, "Let us make man in our image," enabled him to harness all the elements of force and draw rein over every law, until the myriad forms of invisible life stand out in full relief under the finely adjusted lenses of his microscope, and by his telescopic power, made him able to reduce the trackless spaces of an universe to such a degree as to make clear their physical contour and reveal even the geography of the heavenly bodies.

Let us consider this sublime revelation of wisdom in the complex workmanship manifested in our physical being, and which is given us especially as physicians for our study and care. It is unnecessary to go beyond the gross anatomy of our frame to become convinced of a supreme wisdom, but when we enter the department of histology, we stand in confusion before the Omniscient. And while the microscopy of the molecule and the cell baffle our magnified vision, what shall we do with the electrical force we call life, which guides and renews these constant mole-

1. Read at the annual meeting of the Medical Association of Central New York, at Rochester, October 20, 1896.

cular changes? But how slow has been the progress of the knowledge of our marvelous mechanism. When we remember that man lived for thousands of years without understanding the circulation of the blood and it was reserved for one almost of our own time to demonstrate this, as well as the functions of many of the vital organs, may we not be justly proud of our present acquirements? But hold: our brilliant results are being so rapidly transformed that almost ere they are applied, our neighbors, or perhaps we ourselves, have improved upon or so modified our work as to suggest wonderment in our own minds; and while the populace applaud results, the scientist laughs at the same as crude and unworthy, in view of the latter development; and we may well ask, What of the future?

It is generally expected of our profession, which in its research comes nearer than any other to the great first cause, that we not only exercise the healing and curative art, but that we guard our clients from the inroads of disease, and prevent the development in their systems of those germs of decay and death which are now known to swarm upon the mucous surfaces, and in the cutaneous pores, only waiting such conditions as conduce to their pathogenic action or that of their ptomaines; and today we are recognised as occupying that position of trust which rightfully belong to us, only when we intelligently comprehend and use the advantages we have in this realm of scientific investigation.

It is in the field of bacteriology and microscopy that our means of prevention is chiefly centered, and where we verify or correct what our gross finding has shown to be a probable diagnosis. The chemical and microscopical examination of the secretions and excretions, the bacteriological culture of the exudative products of toxic action, and the attendant serum therapy of the present time, all testify to the studious and untiring labor of many who have stood out conspicuously in these later years, and it is quite probable that the close of the nineteenth century will witness the ability on the part of the better equipped physician and surgeon to take from the list of incurable maladies many diseases which to have contracted in time past was to abandon all hope of else but more or less speedy death, and that often in its most repulsive and painful forms.

It appears to the writer that at no very distant day we may shelve in our laboratories each and every coccus and bacillus productive of the long train of toxic disturbances of the human system, labeled with its respective antitoxin, as we now have displayed in our museums monstrosities and malignant tumors which have brought death to so many victims. Writing from a surgical point of view, it seems that the appointments of the modern operating room are the embodiment of aseptic and antiseptic perfection, but I venture the suggestion that these, with the complete technique of a McBurney or a Weyth, may be thought clumsy when compared with what the student of surgery of the twentieth century will be taught. May we not reasonably predict, that should another war blight our fair country the equivalent of so many limbs sacrificed in the late rebellion would be saved to the comfort and use of thousands who might sustain wounds similar to those received in that great struggle; and this, not because we understand anatomy better, but because we have dived down into an under-current of science and brought up treasures of wisdom that enable us to neutralise so many of the germs of infection, fermentation and putrefaction? Limbs that were formerly amputated, because there was no time to properly dress them, will be saved, because, forsooth, it would waste valuable time to amputate. Such will be the perfection of army surgery in the next war.

And as we admire the present status of practical medicine, and congratulate ourselves on our diagnostic appliances and the ease with which we meet the indication with tablet triturate of the ultimate alkaloid, the use of lavage, enteroclysis, massage and electrolysis, we may have to relegate these boasted achievements into innocuous desuetude, and go about our exhibition of the remedial measures of the coming decades, with wonder that we so long drank from the gourd of empiricism, when the golden goblet of a purer science was so nearly within our reach. But have we not lost some ground in so generally dropping some valuable means of relief? It is the judgment of many advanced teachers, that some of the old-time and wellnigh abandoned means of therapy should again be brought into more general use, viz.: venesection, vesication and the seton.

In the field of obstetrics, where those of us who were at work a third of a century ago, went to our task unwittingly the bearers of germs of infection, we now save our parturient patient from all such danger, and where sapremia and septicemia with their suffering, and death followed in a substantial percentage, we now almost never encounter these conditions and can feel the greatest assurance that when we can control the technique of accouchment and the puerperium, the occurrence of fever to any serious degree, has no passport into the lying-in chamber. And more than this, when our fair patient places herself in the care of the scientific obstetrician during the parturient, he may, by intelligent antepartem investigation, so predict the on-coming climax of gestation, as to assure her, as well as himself, that all the physical conditions of the system at large, as well as the functional work of the excretory organs, are favorable or otherwise, and if otherwise, prepare for whatever condition is found to exist, so greatly reducing the risk of mortality to both mother and child.

It is well here to remind ourselves that we are this year celebrating the centenary of the discovery of vaccination, and we are now only four days past the semicentenary of the discovery of anesthesia. Dr. Morton, who though the first to practically apply anesthesia, may have to divide the honor of its first conception with Dr. Colton, who about the same time demonstrated the efficiency of nitrous oxide and Dr. Simpson that of chloroform. Dr. Morton's application of ether was, however, earlier and has become far more general in its use. There should be a more fitting commemoration of this than has been made, and all nations should express as well as feel the sentiment of laudation and gratitude. "Without exaggeration it may be said that no more beneficent discovery has ever been made in the healing art, and not more than two or three comparable with it."

Far back in the history of our profession, a crude application of electricity was made in the work of some, but the immediate past is the only epoch in which any considerable results have been realised. I trust you will all join with me in giving honor also to one now living, a son of the illustrious discoverer, whose praises we have just sung. I refer to Dr. W. J. Morton, now in the faculty of the Post-Graduate Medical School and Hospital of New York City, whose department is that of electro-therapy.

The private office or public dispensary which does not contain more or less of electrical equipment is incomplete and at a disadvantage in the progress of modern medical science. But the specialist, not only in neurology and neurasthenia, but as well in laryngology, rhinology, dermatology and gynecology, is unarmed if his armamentarium lacks a static, a cautery, a primary and secondary faradic and galvanic current, regulated and measured by the most improved rheostat and milliampèremeter, and the subject of this paragraph has perhaps done more than any one else to bring on this helpful progress.

I could not justly close this résumé of the advances of our beloved science without reference to the trained nurse and all that that important personage means to hospital and private practice.

From a Florence Nightingale, with her unselfish devotion to the suffering soldier of the Crimean war, down through later years to the scholarly and accomplished graduate of our own cherished schools, we may see a development of scientific advancement along a line infinitely helpful to the work of the modern physician and surgeon. There is also a gradual and acceptable entrance into our ranks of well-trained women who are serving a most wise and useful field in our profession. They are among the most loyal of the disciples of Esculapius, and almost without exception worthy and competent.

Again, the development of the hospital has made conspicuous many life-saving environments of the sick and helpless. The application of sunlight and free ventilation, as well as the more correct idea of rest and exercise, have kept pace with advanced practice of cleanliness, as related to patient and surroundings, as well as to the doctor himself. This development of sanitation and hygiene, together with anesthesia and asepsis, has made it possible to accomplish desired results with a moiety of the drugs formerly used, as well as far less frequent use of catling and saw. Still there is much room for improvement. The application of scientific principles should be made more directly to the work of our boards of health and sanitary commissions, which cannot be done until they are constituted, at least in the majority, of men not only of recognised judgment and influence, but who have been educated along the lines of scientific medicine. We all rejoice that so wise and fair a code has been framed and made a law, regarding the practice of the professions of medicine, pharmacy and dentistry, and we may well encourage a similar course of legislation regarding those placed in authority in the province of hygiene and health. There is also urgent need of more stringent laws governing the work of midwives, to whom, in our cities, is chargeable the loss of much life, as well as many cases of invalidism in the mother and hopeless blindness in the offspring.

There are some untoward tendencies also arising from a lack of appreciation of the scope of our duties and privileges. In the matter of the entrance of women into so many of the avocations in which only men formerly found fields of action, there is more room for fear than hope for her sex. In looking for the highest types of physical, moral and intellectual perfection of women, that which comes of health and body, heart and mind, where do we rest our search? Not at the workshop door, as we watch the procession of overworked and underpaid girls taking up their nightly march toward their scantily furnished, cheerless apartments; not in the corridor of the modern office, where we are permitted to pause and look upon the drudgery of young women toiling long hours over the hieroglyphics which they form, and then translate into written correspondence and records under a nervous strain, of which we have little conception; nor in the so-called women's rights club. No. As our minds revert to conspicuous examples of such, we are met with disappointment. There is, however, a field where the perfection of beauty is attained, where a well-balanced training in girlhood, begetting a standard of health in young womanhood, realises a happiness in mature life when transplanted from the protection of the home of the wise parent to the ultimate freedom of the love bondage, husband and wife live over again the joy and peace which is a foretaste of Paradise. Here we may rest our search, having discovered the fountain of youthful beauty, broadening as age advances into that placid river of beautified character, reflected so beautifully from the face of motherhood in the family home, entering which, she charms and sanctifies, and at the end, leaving which, she is crowned with the sublimity of peace, as she bestows the hand of blessing upon children and grandchildren, who are to take up the harmonious refrain she has so joyously sung these many years. This is no fanciful picture. It would be the rule rather than the exception, were the unwritten laws governing this matter of home-making more regarded and better obeyed. And to whom more than the trusted physician should the people look for counsel and instruction in this. If we are the almoners of these laws, let us look for some of the factors militating against their being understood and applied.

Can we not find it in the morbid and unnatural desire of our young ladies to be independent? They take positions which require but little special training at a lower rate of pay than is just, thereby creating a competition which the brother or father is unable to meet and give support to his dependents, and to supplement his lessened salary, the girls have to train themselves to take higher positions, where they may, by still more increasing the unhealthy competition, temporarily aid in the home support. So, cause becoming the effect and effect cause, the drift is downward, home is deranged, the natural beauty born to the girl is faded and the woman comes forth lacking in the charms she is herself so much responsible for having lost. But the fault is not all on this side. Our fathers in the struggle laid upon them in founding and sustaining a home or amassing a fortune, grasp every opportunity to get the work of the factory or counting-room done by those who will do the same work for less pay, and so the wrong grows worse, fostered by ambition on the one hand and covetousness on the other.

There are, however, I believe an increasing number of noble women who stand at the home altar, and in God's appointed way administer the sacred rights of her sex, receiving the loyal protection and support of loving and trusting husband and son, and one of the most binding duties of a profession, admitted nearer to the very heart of the home than the holy ministry itself, is to nurture this highest of all beauty, the beauty born of a true and pure domestic life.

I would not be understood as condemning the effort of self-support on the part of young women so placed in the providence of God as to make necessary their devotion to wage earning. There are in the circle of our acquaintance such exceptions, and we should encourage and sustain them in their work.

Another fruitful cause of many miscarriages in the economy of home, is the waywardness of so many of our young men. A false estimate of what constitute an ability to maintain a home discourages their hopes, and their should-be savings are spent on companions, who too often encourage and even expect such costly attentions. These young men in a considerable proportion drift downward to the base and immoral substitute for marriage and home and bring to shame and ill-fame recruits for the habitations of sin. This moralising may seem irrelevant to my title, but I am persuaded that such matters fall justly within "modern medical science in its most practical aspects."

We used to wonder, as boys, why in college nomenclature the end of our curriculum was called "commencement," but on reaching this seeming end of student days we found ourselves only just ready to commence the real work of investigation and research, and instead of maturing the ripe fruits of wisdom then, we had only set our scions into the stock of the tree of knowledge and begun to feel the potency of the oncoming and life-giving stream which would make possible our enlargement into men and women who should step forward into the station meant for us when the Almighty proclaimed that the last work of His creation was very good.

18 William Street.

